# Comparison of lung image quality between CT Ark and Brilliance 64 CT during COVID-19

**DOI:** 10.1186/s12880-021-00720-2

**Published:** 2021-12-13

**Authors:** Gonghua Dai, Jiying Duan, Liang Zheng, Miao He, Yanshan Dai, Mingming Zhang, Shuguang Chu

**Affiliations:** 1 Department of Radiology, East Hospital, Tongji University, No. 150, Jimo Road, Pudong New District, Shanghai, 200120 China; 2 Research Center for Translation Medicine, East Hospital, Tongji University, Shanghai, 200120 China; 3 China International Emergency Medical Team, Shanghai, 200120 China

**Keywords:** COVID-19, The shelter hospital CT, Image quality evaluation, CT Ark, Brilliance 64 CT

## Abstract

**Aim:**

This study is to compare the lung image quality between shelter hospital CT (CT Ark) and ordinary CT scans (Brilliance 64) scans.

**Methods:**

The patients who received scans with CT Ark or Brilliance 64 CT were enrolled. Their lung images were divided into two groups according to the scanner. The objective evaluation methods of signal-to-noise ratio (SNR) and contrast-to-noise ratio (CNR) were used. The subjective evaluation methods including the evaluation of the fine structure under the lung window and the evaluation of the general structure under the mediastinum window were compared. *Kappa* method was used to assess the reliability of the subjective evaluation. The subjective evaluation results were analyzed using the Wilcoxon rank sum test. SNR and CNR were tested using independent sample *t* tests.

**Results:**

There was no statistical difference in somatotype of enrolled subjects. The Kappa value between the two observers was between 0.68 and 0.81, indicating good consistency. For subjective evaluation results, the rank sum test P value of fine structure evaluation and general structure evaluation by the two observers was ≥ 0.05. For objective evaluation results, SNR and CNR between the two CT scanners were significantly different (P<0.05). Notably, the absolute values ​​of SNR and CNR of the CT Ark were larger than Brilliance 64 CT scanner.

**Conclusion:**

CT Ark is fully capable of scanning the lungs of the COVID-19 patients during the epidemic in the shelter hospital.

## Introduction

In the Coronavirus Disease 2019 (COVID-19) epidemic in Wuhan, which began in December 2019, there was a human-to-human transmission phenomenon [1, 2]. In order to block the spread of this infectious disease, the Chinese government has built a large number of shelter hospitals in the epidemic area to isolate and treat these patients with COVID-19. Among the COVID-19 patients, 99.3-100% of the patients had abnormal lung computed tomography (CT) scans [3, 4]. Therefore, shelter hospitals were equipped with CT scanners [5]. This kind of CT scanner is called the shelter hospital CT. However, it is different from the ordinary CT fixed in the room. The shelter hospital CT is placed in the containers, which is very fast and convenient to install and transport. However, whether its image quality can meet with the diagnostic requirements for the COVID-19 epidemic is worth investigating.

Generally, for the clinical image quality evaluation of CT scan, objective evaluation method and subjective evaluation method are used [6, 7]. Because the scan details and reconstruction methods of shelter CT and ordinary CT are different, it may cause differences in results [8]. Thus, in this study, both methods were used to clarify the differences in image quality between the shelter hospital CT (CT Ark) and ordinary CT (Brilliance 64 CT).

## Materials and methods

### CT scanner

The shelter hospital CT was CT Ark (MinFound Medical Systems Co., Ltd; Shaoxing, China). The ordinary CT was Brilliance 64 (Philips Healthcare; Cleveland, OH, USA). The scan parameters and reconstruction details are listed in Table 1.

**Table 1:** Scan and reconstruction details of the two CT scanners

Scanner	Scan details					Reconstruction details			
	Detector type	kV	Rotation time	mA	Pitch	Algorithm	Thickness	Gap	Iterative reconstruction
CT Ark	16 × 1.16 mm	120	0.75 s	100	1.5	Lung	1 mm	1 mm	NDI level 3*
						Standard	5 mm	5 mm	NDI level 3*
Brilliance 64	64 × 0.625 mm	120	0.5 s	Self-adaption	0.798	Lung	2 mm	1 mm	Without
						Standard	3 mm	1.5 mm	Without

*NDI is the product name of iterative reconstruction method of CT Art and level 3 is the iterative weighting level

## Ethics

The study was approved by the Examination of Human Biomedical Research Ethics committee of Shanghai East Hospital Affiliated to Tongji University (Approval No.: [2020] JSR No. (098)). Due to the retrospective nature of the study, informed consent was waived. We confirm that all methods were carried out in accordance with relevant guidelines and regulations. We confirm that all experimental protocols were approved by Examination of Human Biomedical Research Ethics committee of Shanghai East Hospital Affiliated to Tongji University.

## Patients

A total of 122 patients who were scanned from 2020.02.13 to 2020.03.12 with the CT Ark and 122 patients who were scanned from 2020.02.25 to 2020.12.31 with the Brilliance 64 were randomly selected. In order to avoid the effect of the disease itself on image quality, the patients of Brilliance 64 were suspected of viral pneumonia with Radiology diagnosis. The sex and age of patients were recorded to further make sure whether there are different somatotypes between different scanner groups, so that the impact on image quality evaluation was considered [9]. Identification of somatotype was done by measuring Effective Diameter [10]. Specific measurement method [11] and calculation formula (1) were implemented according to the method described in the American Association of pharmaceuticals in medicine (AAPM) report NO. 204 [12].

1 $$D_{{eff}} = \sqrt {D_{{AP}} \cdot D_{L} }$$

The *D*_*eff*_ is the effective diameter; *D*_*AP*_ is anteroposterior diameter; and, the *D*_*L*_ is lateral diameter.

## Observation and measurement tools

The image browser was RadiAnt DICOM viewer version 5.5.1 (64 bit) and the browser default window width and window level were used. The JUSHA M-32 medical diagnosis display screen (JUSHA Medical; China, Nanjing) was used as the only tool for observation and measurement.

## Objective evaluation method

**Fig. 1:**
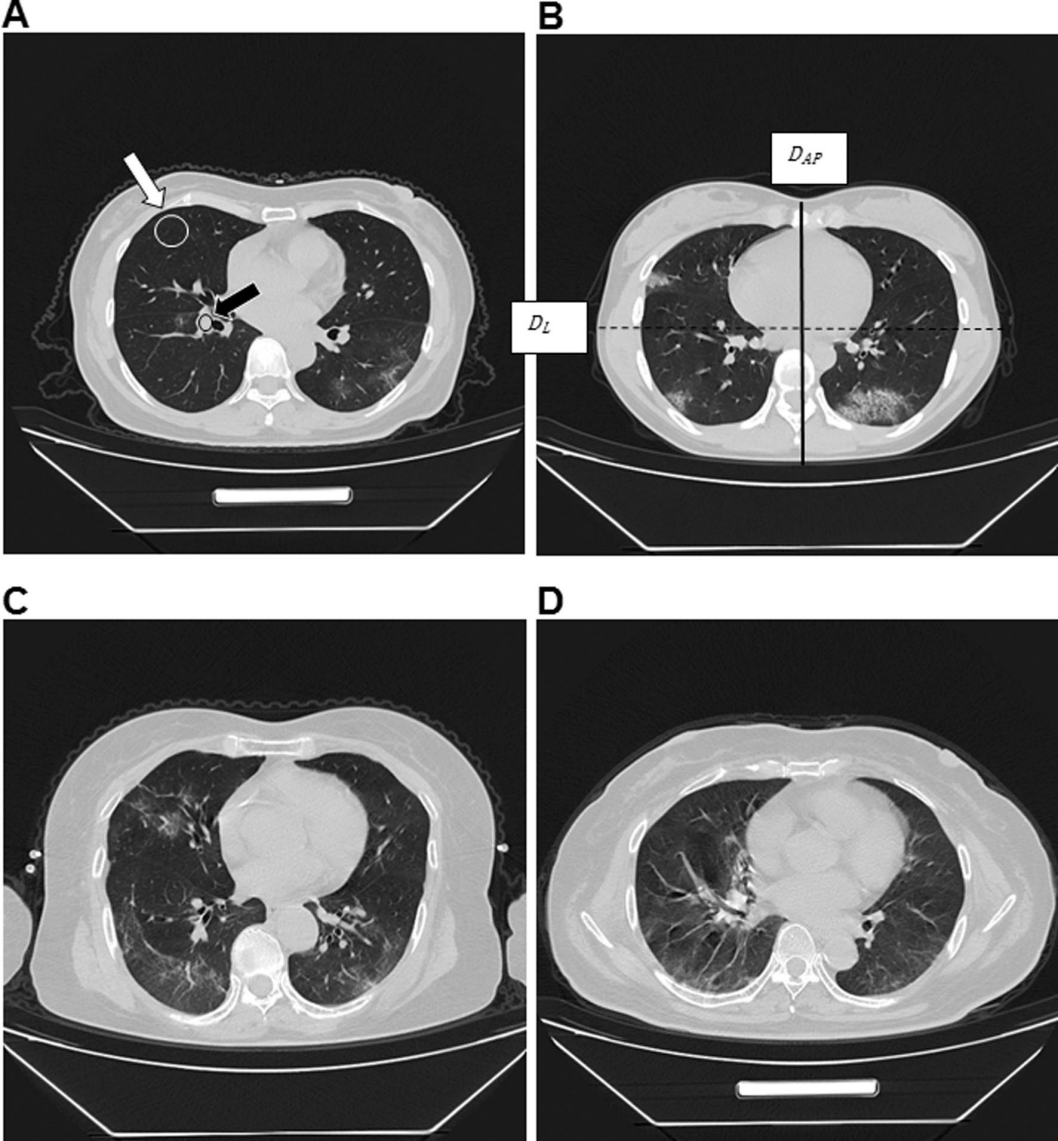
All images were collected from Wuhan shelter hospital. **a** The representative image with the subjective fine structure evaluation of 4 points. The black arrow was the place where the contrast noise ratio was measured in the right lower pulmonary artery, and the white arrow was the place where the contrast noise ratio was measured in the lung field. **b** The representative image with the subjective fine structure evaluation of 3 points. The solid line represents *D*_*AP*_ (anteroposterior diameter), and the dotted line represents *D*_*L*_ (lateral diameter). **c** The representative image with the subjective fine structure evaluation of 2 points. **d** The representative image with the subjective fine structure evaluation of 1 point

Contrast noise ratio (CNR): The disease severity of COVID-19 patients was mainly evaluated by observing the texture of lungs. Therefore, the CNR measurement of the lung texture and the lung field was selected. When the lung window setting is selected, the layer of the proximal right lower pulmonary artery was the standard layer. Using an ROI (region of interest) of ​​50mm^2^-100mm^2^, the right lower pulmonary artery (black arrow in Fig. 1a) and the lung field with sparse lung texture (white arrow in Fig. 1a) were measured. The CT values ​​and standard deviation (SD) were measured twice and the average values were used. The CNR was calculated according to the following formula: CNR = (HU of right lower pulmonary artery-HU of lung field)/SD [13–15]. SD was the average of the SD of the right lower pulmonary artery and the SD of the lung field.

**Fig. 2:**
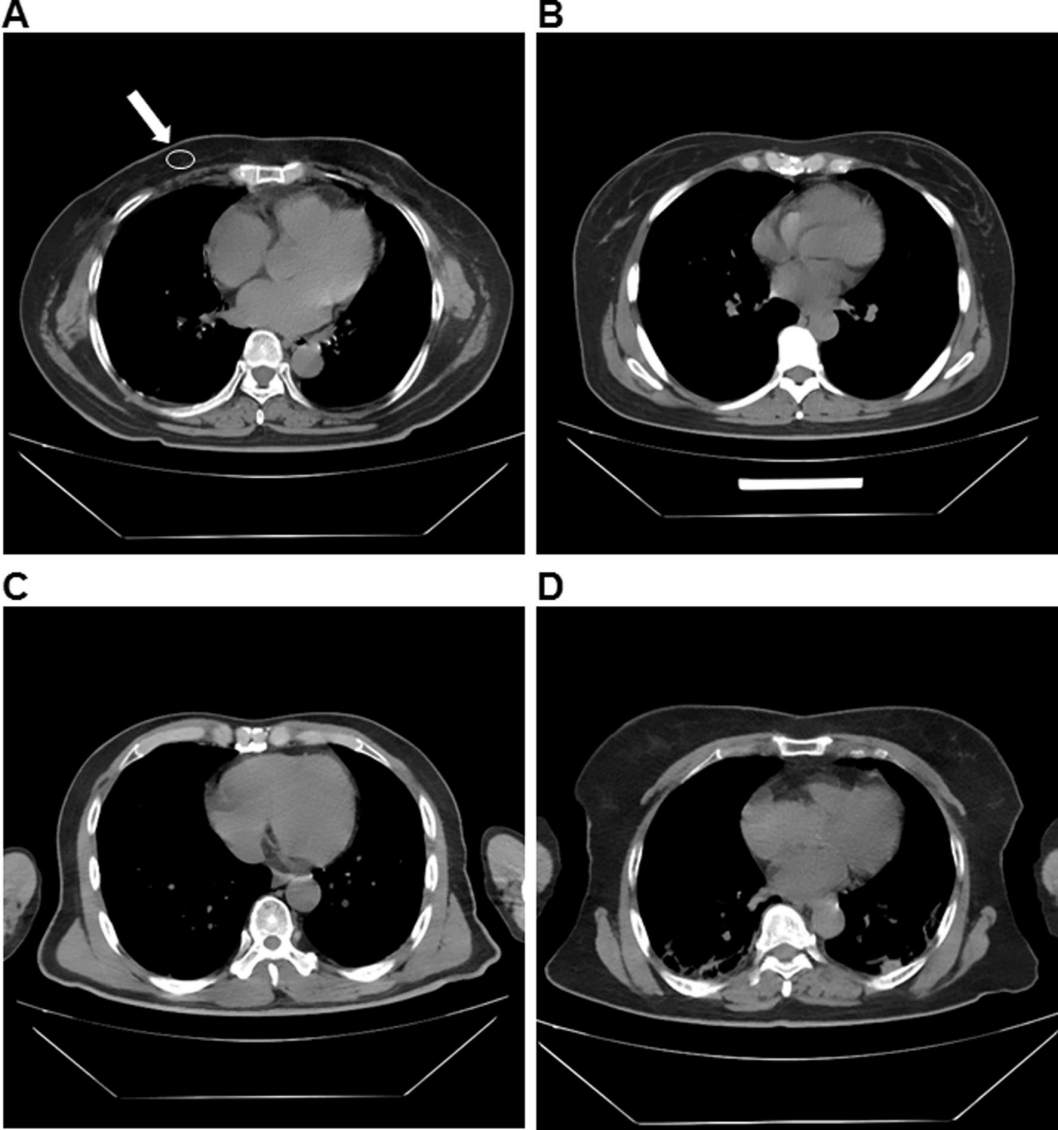
All images were collected from Wuhan shelter hospital. **a** The representative image with the subjective general structure evaluation of 4 points. The white arrow indicates the signal-to-noise ratio of the right anterior chest wall subcutaneous fat measurement. **b** The representative image with the subjective general structure evaluation of 3 points. **c** The representative image with the subjective general structure evaluation of 2 points. **d** The representative image with the subjective general structure evaluation of 1 point

Signal-to-noise ratio (SNR): Considering the large difference in CT value of different tissues, the subcutaneous fat SNR measurement was selected. When the mediastinum window setting is selected, the maximum cross section of the left ventricle was the standard layer. The area of ROI was set as 60mm^2^-250mm^2^ to measure the subcutaneous fat in the right anterior chest wall (white arrow in Fig. 2a). The CT value and SD were each measured twice, and the average value was calculated. The SNR was calculated according to the following formula: SNR = CT value/SD [15].

## Subjective evaluation method

According to the EU standards for CT [17], two observers (A and B), who had more than 15 years of experience as radiologist, observed the fine structure by the lung window and the general structure by the mediastinum window of image in a blinded manner. The images were evaluated according to the 4-point method, which was slightly different from the EU standard according to the actual situation.

Evaluation of fine structure: 4 points were defined as clear display of fine structure with excellent contrast, sharp and clear edges of lung texture; 3 points were defined as display of fine structure with good contrast, slight artifacts, and blur of lung texture; 2 points were defined when the structure was slightly blurred with artifacts in the lung texture and lung field, but the image could be used for diagnosis; 1 point was defined when the fine structure was not clear, the lung texture was blurred, artifacts were more often observed in the lesion, and the image could be barely be used for diagnosis (Fig. 1 a–d).

Evaluation of the general structure: 4 points were defined as clear display of the general structure with clear muscle and fat boundaries; 3 points were defined as display of the general structure, clearer boundaries of muscle and fat, and uneven internal muscle density; 2 points were defined when the structure was shown but the muscle and fat boundary was blurred with artifacts on the edges of bones or muscles; 1 point was defined when only the general structure was displayed, the details in muscles and fats were blurred, and there were more serious artifacts that may affect observation (Fig. 2a–d).

### Statistical analysis

All statistical analyses were performed using SPSS software (Version 19.0, IBM SPSS Statistics, USA, NY, Armonk). The age and Effective Diameters of all patients were grouped according to scanners, and independent sample t-test was performed. Chi-square test was used to analyze the sex distribution difference between the two groups. To determine the reliability of the subjective evaluation method, the *Kappa* method was used to analyze the consistency of the fine evaluation and general structure evaluation data of the two observers. The *Kappa* coefficient was interpreted as follows: ≥0.81 indicating excellent, 0.61–0.80 substantial, 0.41–0.60 moderate, 0.21–0.40 fair, and ≤ 0.20 poor agreement [18, 19]. The subjective evaluation results of the two CT scanners were analyzed using the Wilcoxon rank sum test. The SNR and CNR of the two CT scanners were tested using independent sample *t* tests. *P* <0.05 was considered statistically significant.

## Results

### Baseline characteristics of patients

There was no statistical difference in Effective Diameter and sex between two groups (Table 2). However, there was significant difference in age between the two groups. The difference may be due to the fact that the patients admitted to the shelter hospital were mostly younger patients with mild COVID-19.

**Table.2:** Comparison of Effective Diameter, age and sex of two groups

Group	Male (%)	Female (%)	Total (%)	Age (years)	Effective diameter (cm)
CT Ark	64 (52.5%)	58 (47.5%)	122 (54.5%)	50.33±12.49	24.11±2.77
Brilliance 64	69 (56.6%)	53 (43.4%)	122 (45.5%)	61.21±16.16	23.74±1.15
χ^2^	–	–	0.413	–	–
*t*	–	–	-	− 5.89	1.34
*P*	–	–	0.607	0.01	0.181

The sex differences were analyzed by Chi-square test. The age and Effective Diameter were analyzed by independent sample t-test

### ***Kappa*** test of subjective evaluation results between two observers

*Kappa* test showed that the consistency of two observers in subjective fine structure evaluation of CT Ark was *Kappa *= 0.75 (*P*<0.01), and that in subjective general structure evaluation of CT Ark was *Kappa *= 0.77 (*P*<0.01). For subjective fine structure evaluation of Brilliance 64, the consistency showed that *Kappa *= 0.81 (*P*<0.01); and, for subjective general structure evaluation of Brilliance 64: the consistency *was Kappa*=0.68 (*P*<0.01). The subjective evaluation results of both observers could reach substantial agreement. The results suggest that the subjective evaluation results of the two observers have good consistency and that the subjective evaluation method is reliable and feasible.

## Subjective evaluation results

In the subjective evaluation results of the shelter hospital CT by CT Ark and ordinary CT by Brilliance 64 of the two observers, there was no statistical difference between the two groups (*P*>0.05). The mean rank of the general structure and the fine structure was roughly equal (Table 3).

**Table.3:** The rank sum test results about two observer’s subjective evaluation results

Group	The mean rank of observer A		The mean rank of observer B	
	Evaluate of fine structure	Evaluate of general structure	Evaluate of fine structure	Evaluate of general structure
CT Ark	124.50	128.32	126.85	118.53
Brilliance 64	120.50	116.68	118.15	126.47
*Z*	− 0.47	− 1.40	− 1.02	− 0.96
*P*	0.64	0.16	0.31	0.34

## Objective evaluation results

In the pair-wise comparison of the SNR and the CNR, the results showed significant statistical differences between the two groups (Table 4). It is worth noting that the absolute values ​​of the SNR and CNR by CT Ark were larger than those by Brilliance 64.

**Table.4:** The independent-samples T test results about the SNR and CNR of two groups

Group	SNR mean value	CNR mean value
CT Ark	− 15.44±4.75	24.45±5.63
Brilliance 64	− 8.74±2.64	10.68±3.37
*t*	13.63	− 23.18
*P*	0.00	0.00

SNR, signal-to-noise ratio; CNR, contrast noise ratio

## Discussion

In this global pandemic of COVID-19, run-up of medical resources and management of infected patients is a practical problem that we urgently need to solve. Among them, building shelter hospitals for treatment of mild patients, and avoiding the spread of virus in family are very important [5]. Shelter hospital CT plays an important role in the diagnosis of COVID-19 [20]. In the present study, we assessed the image quality of shelter hospital CT by CT Ark and ordinary CT by Brilliance 64. Both the subjective evaluation and the objective evaluation results revealed that the image quality by CT Ark and by Brilliance 64 were consistent, indicating that CT Ark is fully capable of scanning the lungs of the COVID-19 patients during the epidemic in the shelter hospital. Our findings provide evidence for the reasonable application of shelter hospital CT (CT Ark).

According to our practical experience, the shelter hospital CT has better mobility than the ordinary CT. The ordinary CT has a large volume, long installation, and commissioning time, high requirements on site, ventilation, temperature, and humidity, etc., and cannot be carried out in the wild. The vehicle borne CT has small space and low machine power. It is mostly used for head scanning. However, the whole body scanning cannot be completed. Moreover, the distance from the ground to room is long. Some patients are not convenient to get on and off, and it is not convenient for the transfer bed to enter and exit. The shelter hospital CT inherits the flexible, modular, and emergency features of the shelter hospital. While having full feature of CT, it also tries to reduce the volume as much as possible and optimize the structure. The shelter hospital CT can be transported to the destination by a container truck, and it can be used after being hoisted. The operating room and the scanning room are not directly connected to each other, and they are installed with independent exhaust fan and air conditioner. The operating room is equipped with air sterilizer and hand disinfection equipment. The scanning room is equipped with remote control ultraviolet light. The technician can control the switch of the ultraviolet light through the remote control in the operating room. It is beneficial for infection isolation and emergency use [21].

Previous studies have shown the role of imaging and shelter hospital construction in blocking the transmission of COVID-19 and patient treatment evaluation [5, 20, 22]. However, the image quality of shelter hospital CT is not well evaluated. Therefore, we collected some patient images from the shelter hospital CT for comparison with the ordinary CT. First, there was no difference in the somatotypes between patients of the two CT scanner groups. Thus, we can exclude the image quality difference caused by the somatotype difference. Then, in the subjective evaluation, there was no statistical difference between two groups in the fine structure evaluation and the general structure evaluation. Because the subjective evaluation of image represents the actual experience in clinical use, our quality comparison is still based on the subjective evaluation.

In the objective evaluation, the scanning and reconstruction details of these two scanners were inconsistent. Therefore, the results of the comparison were different. It is worth noting that the absolute values of the SNR and CNR of the CT Ark were larger than those of Brilliance 64. This means that either the CT value of CT Ark is higher, or its SD value is lower. From the result of measurement, this phenomenon was indeed caused by the lower SD value of the CT Ark. This may be related to the iterative reconstruction method used by CT Ark. Iterative reconstruction can effectively reduce the SD value and increase the SNR [6, 23–25], which can improve the image quality of shelter CT.

This study has some limitations. First, the sample size is relatively small. Second, only the images of patients with mild COVID-19 were assessed. Third, only two brands of CT scanners were included for assessment. Further studies, such as those with larger sample sizes, are warranted to confirm our results.

In summary, from the results of subjective observations, there is no difference in the image quality of the shelter hospital CT by CT Ark and ordinary CT by Brilliance 64. Therefore, the shelter hospital CT of CT Ark is fully capable of scanning for COVID-19. The shelter hospital CT provides a more convenient and effective implementation method for the blocking and control of the COVID-19 epidemic.

## Data Availability

The authors confirm that the data supporting the findings of this study are available within the article.

## Abbreviations

COVID-19: Coronavirus Disease 2019
CT: computed tomography
CNR: contrast noise ratio
SD: standard deviation
SNR: signal-to-noise ratio
ROI: region of interest
